# Generation of femtosecond γ-ray bursts stimulated by laser-driven hosing evolution

**DOI:** 10.1038/srep30491

**Published:** 2016-07-26

**Authors:** Yong Ma, Liming Chen, Dazhang Li, Wenchao Yan, Kai Huang, Min Chen, Zhengming Sheng, Kazuhisa Nakajima, Toshiki Tajima, Jie Zhang

**Affiliations:** 1 Beijing National Laboratory of Condensed Matter Physics, Institute of Physics, CAS, Beijing 100190, China; 2 IFSA Collaborative Innovation Center, Shanghai Jiao Tong University, Shanghai 200240, China; 3 Institute of High Energy Physics, CAS, Beijing 100049, China; 4 Department of Physics and Astronomy, Shanghai Jiao Tong University, Shanghai 200240, China; 5 Department of Physics, Scottish Universities Physics Alliance, University of Strathclyde, Glasgow G4 0NG, United Kingdom; 6 Center for Relativistic Laser Science, Institute for Basic Science (IBS), Gwangju 500-712, Republic of Korea; 7 Department of Physics and Astronomy, University of California, Irvine, California 92697, USA

## Abstract

The promising ability of a plasma wiggler based on laser wakefield acceleration to produce betatron X-rays with photon energies of a few keV to hundreds of keV and a peak brilliance of 10^22^–10^23^ photons/s/mm^2^/mrad^2^/0.1%BW has been demonstrated, providing an alternative to large-scale synchrotron light sources. Most methods for generating betatron radiation are based on two typical approaches, one relying on an inherent transverse focusing electrostatic field, which induces transverse oscillation, and the other relying on the electron beam catching up with the rear part of the laser pulse, which results in strong electron resonance. Here, we present a new regime of betatron γ-ray radiation generated by stimulating a large-amplitude transverse oscillation of a continuously injected electron bunch through the hosing of the bubble induced by the carrier envelope phase (CEP) effect of the self-steepened laser pulse. Our method increases the critical photon energy to the MeV level, according to the results of particle-in-cell (PIC) simulations. The highly collimated, energetic and femtosecond γ-ray bursts that are produced in this way may provide an interesting potential means of exploring nuclear physics in table top photo nuclear reactions.

Two kinds of laser-driven synchrotron X-ray sources based on laser plasma acceleration have been studied extensively^1^. One method exploits a quasi-monoenergetic electron beam generated via laser wakefield acceleration^2^ to drive an external permanent magnet undulator for the generation of soft X-rays^3^. In this case, the plasma serves as an accelerator and the undulator induces transverse oscillation, typically resulting in the radiation of ~1 keV photons with a narrow spectral bandwidth from an undulator with a 1-cm period driven by a 1-GeV electron beam^4^. The other method relies on the plasma wake excited by an intense laser pulse, which serves as both accelerator and wiggler^5^. This latter method may have an advantage in producing a wide range of energetic photons from the X-ray region to as far as the γ-ray region^5^,^6^,^7^. Moreover, the simplicity and compactness of such a coupled accelerator/wiggler system make it possible to develop a tabletop-scale synchrotron radiation source^1^,^8^. In the bubble regime^9^,^10^,^11^ of laser plasma acceleration, where nonlinear wakefields on the order of 100 GV/m are excited, subject to a strong focusing force due to the transverse wakefield, electrons that are injected off the axis exhibit periodic transverse oscillations during the acceleration process and consequently emit synchrotron radiation, often referred to as betatron radiation^1^,^5^,^6^,^7^,^12^,^13^. The properties of this betatron radiation are entirely determined by the electron dynamics in the plasma bubble and the Liénard-Wiechert potentials^14^. For example, the critical photon energy of the betatron radiation is given by *E*_*c*_ = *3ħω*_*β*_*Kγ*^*2*^, where *K* = *γω*_*β*_*r*_*β*_/*c* is a parameter describing the strength of the betatron oscillation of an electron with an energy of *mc*^*2*^*γ*, *ω*_*β*_ = (*2γ*)^*−1/2*^*ω*_*p*_ is the betatron oscillation frequency with a plasma frequency of *ω*_*p*_ = (*4πe*^*2*^*n*_*p*_/*m*)^*1/2*^ at the plasma density *n*_*p*_ and *r*_*β*_ is the amplitude of the betatron oscillation. The photon flux at the peak photon energy is given by *N*_*photon*_ ≈ *3.3×10*^*−2*^*N*_*e*_*N*_*β*_*K*, where *N*_*e*_ is the number of electrons and *N*_*β*_ is the number of periods of betatron oscillation^1^. Therefore, a larger betatron amplitude and electron charge lead to a higher photon energy and radiation flux. However, to produce high-energy and high-quality electron beams, the laser spot size *w*_*0*_should be matched with the bubble size *R*_*B*_to ensure the formation of a nearly spherical bubble and achieve stable propagation of the laser pulse under the matching condition^11^of *k*_*p*_*R*_*B*_ ≈ *k*_*p*_*w*_*0*_ ≈ *2*(*a*_*0*_)^*1/2*^, where *k*_*p*_ = *2π/λ*_*p*_is the plasma wave number for a plasma wavelength of *λ*_*p*_ and *a*_*0*_ = *eA*_*0*_*/mc*^*2*^ is the normalized vector potential of the laser pulse for a peak amplitude *A*_*0*_ of the vector potential. Under this condition, if electrons are self-injected into the bubble on the axis, then the transverse wakefield will tend to focus the electron beam rather than induce transverse oscillations. By contrast, for the generation of highly brilliant betatron radiation with higher photon energy, it is interesting to explore how to enhance the betatron oscillations and the beam charge. In this context, the enhancement of the betatron amplitude can be manipulated to some extent by exploiting the forced oscillation of the self-injected electron beam in the plasma bubble^15^, where the betatron oscillation is resonantly driven by the laser field, leading to the extension of the spectral peak photon energy up to ~150 keV^7^. However, this technique for exciting resonant betatron oscillations is inefficient in enhancing the charge and betatron amplitude of an electron bunch with an energy spread, resulting in limited peak brilliance of the betatron radiation.

In this paper, we present an efficient method for stimulating large-amplitude transverse oscillations of an electron beam to produce betatron radiation with a spectral peak energy near 1 MeV. With laser energy around 10 J and suitable plasma parameters, a large quantity of electrons can be continuously injected into a longitudinally stretched bubble and accelerated up to a multi-GeV energy level. These bunch exhibit large-amplitude collective transverse oscillations because of strong self-modulation and hosing motion of both the laser pulse and the plasma bubble^16^,^17^,^18^,^19^,^20^,^21^. Because of the large betatron oscillation amplitude and the high average energy of the continuously injected electron bunches, the photon energy of the betatron radiation reaches the γ-ray region, and, furthermore, its peak brilliance may be significantly enhanced because of the increased oscillation amplitude and number of electrons.

## Results

In particle-in-cell (PIC) simulations, a 144-TW laser pulse with *a*_*0*_ = 3.64 and *w*_*0*_ = 18 μm was launched into a homogeneous plasma (see Methods for the detailed simulation parameters). The plasma density was deliberately chosen to be *n*_*p*_ = 2.0 × 10^18^ cm^−3^, slightly higher than the optimal density of 1.3 × 10^18^ cm^−3^ determined from the matching condition, i.e., *k*_*p*_*w*_*0*_ ≈ 2(*a*_*0*_)^1/2^. Such a slightly higher density is likely to cause self-focusing of the laser pulse and lower the self-injection threshold, leading to multiple injections^22^−^28^.

**Figure 1:**
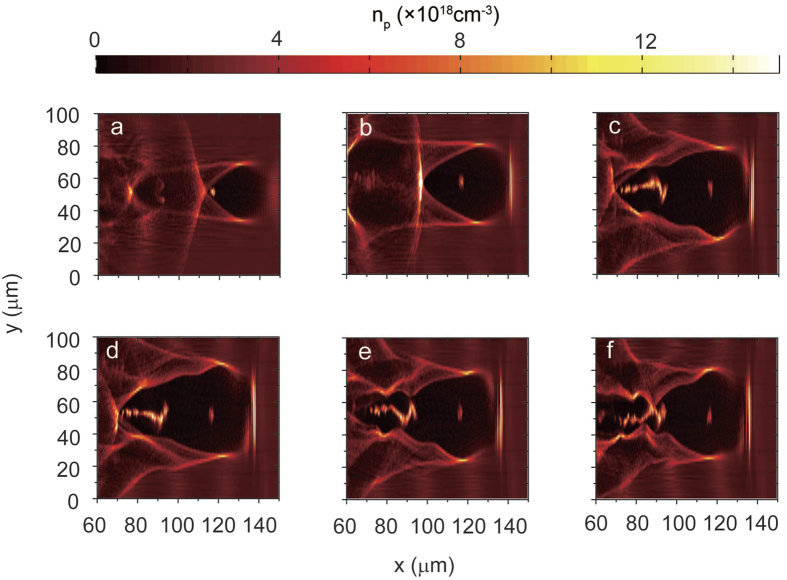
Evolution of the electron density distribution: (**a**) t = 3.3 ps, (**b**) t = 20.9 ps, (**c**) t = 27.5 ps, (**d**) t = 28.4 ps, (**e**) t = 29.0 ps, and (**f**) t = 30.0 ps.

**Figure 2:**
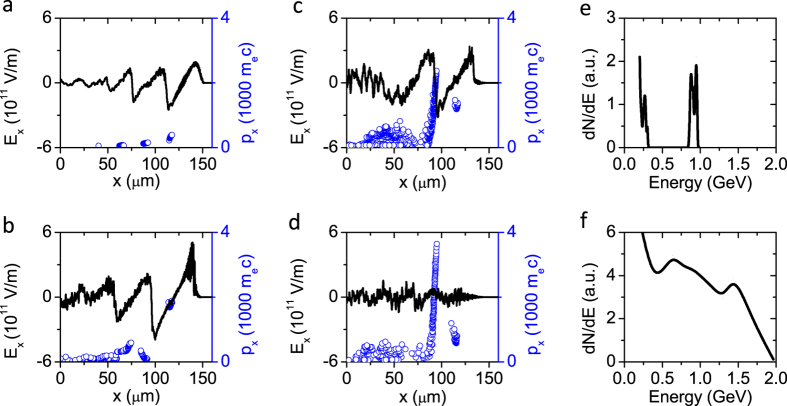
Longitudinal electric field distribution, electron phase space (*x* − *p*_*x*_) and spectra: the blue lines represent the electric field and the black dots represent the electron phase-space distribution at (**a**) t = 3.3 ps, (**b**) t = 20.9 ps, (**c**) t = 36.2 ps, and (**d**) t = 52.0 ps; the first bunch and the second bunch are accelerated to their maximum energies at t = 20.9 ps (**b**) and t = 52.0 ps (**d**) respectively. (**e**) The spectrum of the first bunch at t = 20.9 ps, with a peak energy of *E* ≈ 1 GeV and an energy spread of Δ*E*/*E* ≈ 10%. (**f**) The spectrum of the second bunch at t = 52 ps.

**Figure 3:**
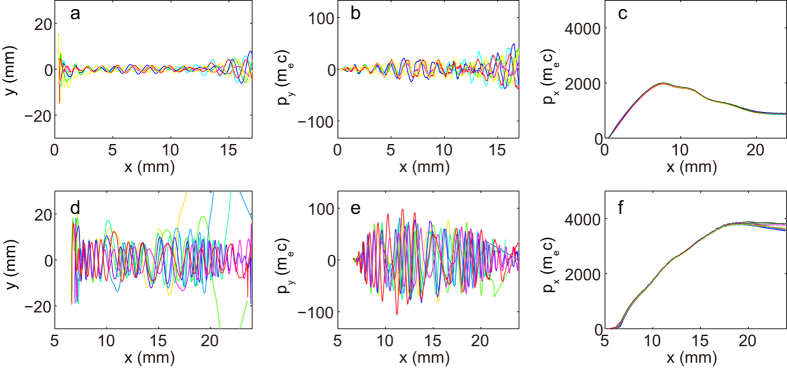
Typical electron behaviors of the two electron bunches. (**a**) The electron trajectories of seven randomly selected particles from the first bunch, indicating that the average betatron oscillation amplitude is r_β_ ~ 1 μm. The corresponding transverse and longitudinal momentum evolutions of these particles are shown in (**b**,**c**), respectively. (**d**) The electron trajectories of seven randomly selected particles from the second bunch, indicating that the average betatron oscillation amplitude is r_β_ ~ 9 μm. The corresponding transverse and longitudinal momentum evolutions of these particles are shown in (**e**,**f**), respectively.

Multiple injections were observed in the 2D PIC simulations, as shown in Figs 1 and 2a–d. At the beginning of the interaction, as shown in Fig. 1a, the laser pulse drives a stable spherical bubble, while self-injected electrons form a tiny single bunch and travel with the betatron motion with a small average amplitude of *r*_*β*_ ≈ 1 μm, as shown in Fig. 3a. After the laser pulse has propagated for 20.9 ps, this bunch is accelerated up to its maximum energy of 0.96 GeV, with an energy spread of Δ*E*/*E* ≈ 10%, as shown in Fig. 2e. Meanwhile, the bubble is significantly stretched in the longitudinal direction and the second injection process begins, as seen in Fig. 1b. Accompanying the onset of injection of the second bunch, almost all electrons in the rear sheath of the elongated bubble avalanche and become trapped, as shown in Fig. 1c. From Fig. 1c–f, one can see that the second electron bunch experiences collective transverse oscillations with an average amplitude *r*_β_ as large as 9 μm, as shown in Fig. 3d (the entire process is illustrated more visually in the Supplementary Information). Figure 2 shows snapshots of the acceleration process of the second bunch in *x*-*p*_*x*_ phase space; simultaneously, the first bunch has already begun to decelerate after *t* = 20.9 ps (*x *= 6.3 mm). Notably, the acceleration of the second bunch lasts more than 30 ps, until the depletion of the laser pulse at *t *= 52 ps (*x *= 15.6 mm). Figure 2f shows the energy spectrum of the electrons in the second bunch with their maximum energy of 1.94 GeV, i.e., two times higher than that of the first bunch; the total number of electrons for energies greater than 200 MeV (*γ* > 390) is an order of magnitude higher than that in the first bunch. Moreover, unlike the first bunch, which undergoes rapid deceleration after dephasing (Fig. 3c), the second bunch maintains its maximum energy for more than 20 ps (Fig. 3f) and simultaneously undergoes transverse oscillation (Fig. 3d), thereby enhancing the generated radiation flux. This occurs because of the quasi-uniform longitudinal wakefield modified by the strong beamloading effect driven by the second electron beam itself after the significant elongation of the bubble, as shown in Fig. 2d. By contrast, in Fig. 2b,c, the longitudinal gradient of the wakefield is quite sharp, resulting in the rapid deceleration of the first bunch after dephasing.

**Figure 4:**
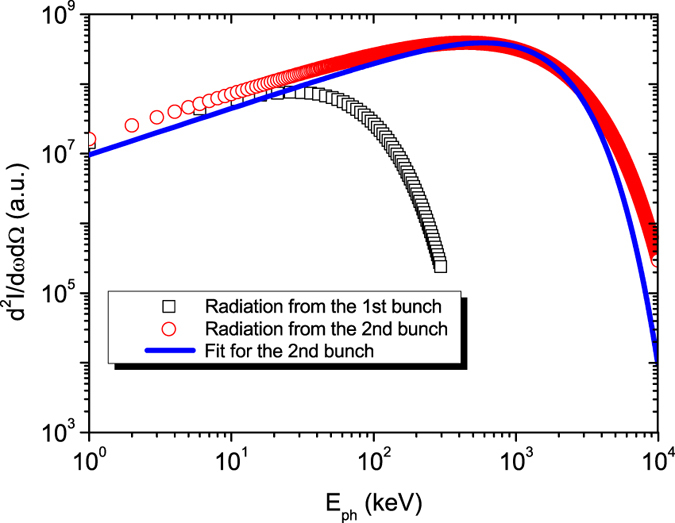
Integrated on-axis spectra of the betatron radiation emitted by the first self-injected electron bunch (black squares) and the second continuously injected electron bunch (red circles), respectively. Blue line represents the best fitting for the on-axis spectrum from the second electron bunch. (See Method for the detailed calculation of the spectra and the critical photon energy of the radiation).

Such a high-energy electron beam with large-amplitude oscillations is very beneficial for the generation of intense betatron radiation. The on-axis spectrum of the betatron radiation is obtained by integrating the contributions from all electrons in the second bunch, as shown in Fig. 4. This integrated betatron radiation spectrum has a critical photon energy of *E*_*c*_ ~ 1.2 MeV and a high-energy tail extending to 10 MeV. For comparison, the spectrum of the betatron radiation emitted by the first bunch, which has a critical photon energy of *E*_*c*_ ~ 80 keV, is also presented in Fig. 4.

## Discussion

**Figure 5:**
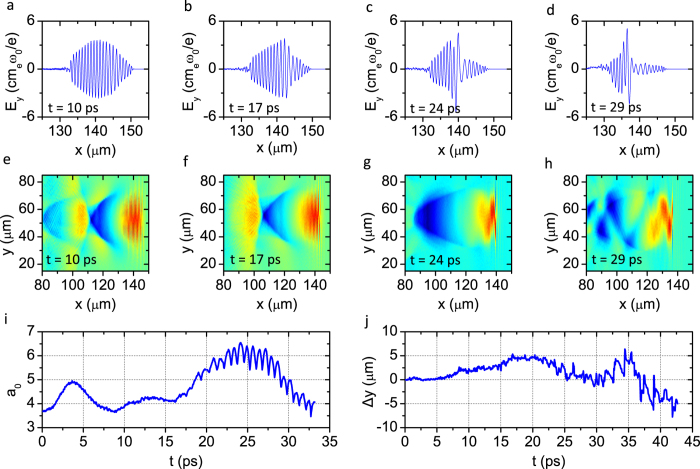
Evolution of the laser pulse and the transverse distribution of the wakefield: snapshots (**a–d**) show the evolution of the laser’s electric field at t = 10, 17, 24 and 29 ps, respectively; snapshots (**e–h**) show the transverse distribution of the longitudinal wakefield at each corresponding time; (**i**) shows the evolution of the laser strength parameter a_0_; and (**j**) shows the deviation of the laser pulse centroid from the x axis. The laser centroid position was defined as the peak position of the transverse distribution of the laser pulse.

We find that the phenomena of bubble elongation and the subsequent electron injection with high charge^26^ and large-amplitude oscillations of continuously injected bunches result from the evolution of the laser pulse (similar laser pulse evolution can also be observed in Fig. 4b of ref. 29, which was generated based on 3D simulations, thereby supporting the rationale behind our 2D simulations). There are two aspects of this evolution: on the one hand, the peak intensity of the laser pulse, 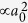, varies, as shown in Fig. 5i, with the periodic self-focusing and defocusing of the laser pulse^29^,^30^; on the other hand, the significant erosion of the laser pulse front results in the asymmetrical distribution of the laser field *E*_*y*_, as seen from Fig. 5a–d. These effects eventually stimulate the hosing^31^ of the plasma wake, as shown in Fig. 5e–h, and the high-frequency oscillations in the laser field strength *a*_*0*_ observed after *t* = 17 ps in Fig. 5i.

Generally speaking, the plasma wavelength increases as the laser intensity increases because of relativistic effects in the plasma response^32^. At the beginning of the interaction, the plasma bubble stretches out and then constricts because of the moderate self-focusing and defocusing of the laser pulse; simultaneously, the first self-injected electron bunch is accelerated in a small phase space volume, reducing its energy spread and transverse beam size. The second injection is triggered by a drastic increase in the laser intensity caused by the significant self-focusing and self-steepening of the laser pulse after *t* = 17 ps, as shown in Fig. 5i. By comparing Fig. 1b with Fig. 1a, one can observe significant stretching of the plasma bubble, leading to the transformation of the bubble geometry from a round to a triangular shape. The bubble structure cannot be sustained when severe distortions occur, eventually leading to the breaking of the first bubble and the second injection. Moreover, because the electrons flowing on the bubble sheath are significantly accumulated in the apex region of the triangular bubble, the local electron density at the rear of the bubble increases from 8 × 10^18^ cm^−3^ at *t* = 3.3 ps (Fig. 1a) to 4.3 × 10^19^ cm^−3^ at *t* = 20.9 ps (Fig. 1b). This local increase in electron density is responsible for the higher charge of the second bunch, which is produced by an avalanche-like breaking of the sheath at the rear of the first bubble.

As seen in Fig. 1c–f, the transverse oscillation and asymmetric deformation of the bubble may cause oscillations of the self-injection region, thereby leading to transverse modulation of the second bunch in conjunction with the betatron oscillation. After t = 17 ps, because of the significant erosion of the laser pulse front, the pulse is shortened to form a sharp front and an asymmetric electric field, eventually resulting in a few-cycle dominant laser pulse, as observed in Fig. 5d. In this situation, a carrier envelope phase (CEP)^33^, referring to the phase shift between the laser pulse and the carrier wave, may play a dominant role in the mechanism of stimulating the hosing motion of the laser and the plasma bubble, as investigated in ref. 34. According to the CEP model of hosing motion, the period of the bubble oscillations can be estimated as *T*_*CEP*_ ≈ (*2πc/ω*_*0*_)(*v*_*p*_*−v*_*g*_)^*−1*^ ≈ *2πω*_*0*_/*ω*_*p*_^*2*^, where *ω*_*0*_ is the laser frequency and *v*_*p*_ and *v*_*g*_ are the linear phase velocity and group velocity, respectively, of the laser pulse in an underdense plasma^34^. This means that the wavelength over the CEP change of 2π is given by *λ*_*CEP*_ ≈ (*n*_*c*_/*n*_*p*_)*λ*_*0*_, where *n*_*c*_ = *mω*_*0*_^*2*^/*4πe*^*2*^ is the critical plasma density. For the simulation at *n*_*p*_ = 2 × 10^18^ cm^−3^, the wavelength of the CEP is approximately *λ*_CEP_ ~ 0.7 mm, corresponding to a period of ~2.3 ps. In Fig. 5j, it is clearly observable that the self-excited oscillations of the laser pulse centroid, with a period of approximately 2 ps after *t* = 21 ps, overlap with the slowly oscillating centroid deviation. However, from Fig. 1c–f, it is not entirely obvious that the bubble oscillations are synchronized with the CEP dynamics. In contrast to the oscillations of the laser pulse centroid, the transverse distribution of the longitudinal wakefield exhibits a transverse oscillation with approximately the linear plasma wavelength *λ*_*p*_ ≈ 24 μm, as shown in Fig. 4h, in addition to the modulation of the bubble sheath, as observed in Fig. 1c–f.

The direct influence of the stimulated hosing motion of the laser and the plasma bubble on the continuously injected electron bunch is that, as shown in Fig. 3e, the transverse momentum of the electrons in this bunch increases dramatically during their acceleration and interaction with the asymmetric wakefield. This increase in the transverse momentum leads to a large oscillation amplitude *r*_*β*_, which eventually boosts the critical photon energy and flux of the betatron radiation. For comparison, as shown in Fig. 3b, the electrons in the first bunch barely gain any transverse momentum during their acceleration in the absence of the asymmetric wakefield because the CEP effect occurs after the dephasing of the first bunch. For both bunches, the initial transverse momentum of the electrons, *p*_*y*_ ~ *m*_*e*_*c*, is determined by the nonlinear ponderomotive force of the laser pulse, ***F***_*pN*_ = *−m*_*e*_*c*^*2*^*∇γ* 31.

**Figure 6:**
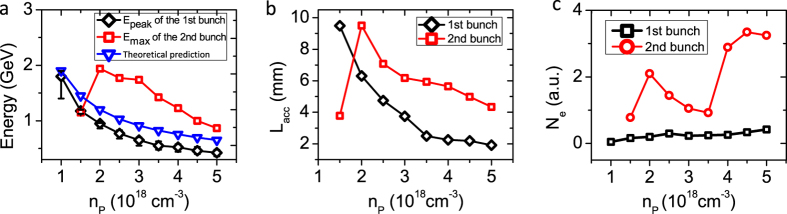
Effects of plasma density on the maximum electron energy, the acceleration length and the total number of electrons: (**a**) The maximum peak energy of the first bunch (black diamonds) and the maximum energy of the second bunch (red squares) as functions of the plasma density. The error bars represent the energy spread of the first bunch. The blue triangles represent the predictions according to the scaling law given in ref. 11. (**b**) The acceleration lengths of the first bunch (black diamonds) and the second bunch (red squares). (**c**) The total numbers of electrons in the first bunch (black squares) and the second bunch (red circles).

Finally, we discuss the plasma density effects. As shown in Fig. 6a, the maximum peak energy of the first quasi-monoenergetic bunch decreases as the plasma density increases. For reference, the bunch energy determined from the simulations was compared with the energy gain indicated by the scaling law, *ΔE* ≈ *mc*^*2*^(*e*^*2*^*P*/*m*^*2*^*c*^*5*^)^*1/3*^(*n*_*c*_/*n*_*p*_)^*2/3*^, for a laser wakefield accelerator driven by a peak laser power *P* at a plasma density *n*_*p*_ in a matched blow-out bubble^11^. As shown in Fig. 6a, the maximum energy of the second bunch is higher than that of the first bunch for plasma densities higher than 2 × 10^18^ cm^−3^, at which density the significant elongation of the first bubble occurs. For example, the maximum energy of the second bunch is ~ 2 GeV at *n*_*p*_ = 2 × 10^18^ cm^−3^, compared with the ~1 GeV peak of the first bunch. Because the longitudinal size of the elongated bubble in which the second bunch is accelerated is apparently larger than that of a single bubble before elongation, the head of the second bunch can undergo acceleration over a longer distance, until the laser pulse energy is completely depleted, unlike in the case of the first quasi-monoenergetic bunch, whose maximum energy is limited by dephasing rather than pump depletion. It is well known from the linear model of laser wakefield acceleration that both the dephasing and the pump depletion length decrease as the plasma density increases, as shown in Fig. 6b. Overall, from a practical perspective regarding the generation of betatron radiation with a higher critical photon energy and a higher peak brilliance, the optimal operating plasma density results in *n*_*p*_ ~ 2 × 10^18^ cm^−3^, considering that the critical photon energy scales as 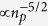 and the peak brilliance scales as 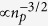, while both higher and lower plasma densities lead to a decrease of the maximum energy of the continuously injected electron bunch, as shown in Fig. 6a, and even no continuous injection occurs at all at plasma density below *n*_*p*_ = 1.5 × 10^18^ cm^−3^.

## Conclusions

In conclusion, a new regime for the generation of betatron γ-ray radiation from laser plasma accelerators is presented. In this regime, a laser pulse with a peak power of 144 TW can drive an electron beam with energy up to 2 GeV and a high-quality quasi-monoenergetic beam with a peak energy of 1 GeV at a plasma density of 2 × 10^18^ cm^−3^. This electron beam can produce femtosecond-duration betatron radiation with MeV-level photon energy. The excitation of a femtosecond MeV γ-ray burst may be understood in comparison with present accelerator-based synchrotron light sources in which X-rays in the range of keV to tens of keV with a 100-ps pulse duration can be produced in electron storage rings of hundreds of meters in circumference, and/or several keV-range fs-scale X-ray pulses can be produced; however, the latter can only be achieved at km-scale linac-based free electron laser facilities such as the LCLS at SLAC, USA and SAKURA at Spring-8, Japan. To produce MeV-range synchrotron radiation γ-rays in the conventional way, a 1-GeV electron beam accelerator and 10-MG bending magnets, which cannot be achieved at present, would be needed. By contrast, our proposed scheme eliminates the need for such a large-scale accelerator facility, requiring only a 2-cm gas-filled plasma accelerator driven by a 100-TW-class laser.

Secondly, our study has focused on investigating the mechanism of production of high-energy electron beam with large-amplitude betatron oscillations, in contrast to many previous works addressing the production of high-quality electron and radiation beams from laser plasma accelerators. The mechanism comprises several complex processes. A relativistic laser pulse with a strength on the order of *a*_*0*_ ~ 4 drives a large-amplitude nonlinear plasma wake under mismatched conditions. The transverse and temporal self-modulation of the laser pulse initially occur in addition to the self-injection and acceleration of electrons as a result of periodic self-focusing and diffraction, and lead to the significant self-compression and erosion of the pulse front. The significant self-steepen of the laser pulse front, on one hand, leads to the significant elongation of the bubble and the continuous injection of the second bunch; on the other hand, results in the CEP effects which induce transverse oscillation and modulation (hosing motion) of both the laser pulse and the plasma bubble and eventually leads to the enhanced betatron oscillation of the continuously injected electron bunch. The continuously injected electron bunch can be accelerated up to GeV with large oscillation amplitude, leading to the generation of enhanced betatron radiation with MeV-level photon energy.

Such a γ-ray source may have broad applications in nuclear physics research concerning photonuclear reactions as well as ultrafast dynamics research in biology and chemistry. The recent advent of compact multi-100-TW-class lasers makes it possible to implement nuclear physics research that exploits such ultrafast γ-ray sources in tabletop experiments in small-scale laboratories.

## Methods

### PIC simulations

The PIC simulations were performed using the 2D PIC code KLAP^35^. A simulation box with a moving window of 100 μm × 160 μm was used, corresponding to 500 × 4000 cells in the y and x directions, respectively, with each cell containing 9 macro particles. The spatio-temporal distribution of the laser pulse can be expressed as *a*(*τ*,*y*) = *a*_*0*_sin(2*πτ*/3*τ*_*0*_)exp(−*y*^2^/*w*_*0*_^2^)cos(2*πτ*/*T*) for 0 <*τ* < 3/2*τ*_*0*_, where *τ* = *t* − *x*/*c*, *a*_*0*_ = 3.64 is the peak amplitude of the normalized vector potential, *τ*_*0*_ = 60 fs is the FWHM pulse duration, *w*_*0*_ = 18 μm is the 1/*e*^2^ spot radius, and *T* = *λ*_*0*_/*c*, with a laser wavelength of *λ*_*0*_ = 800 nm. The intensity of the laser pulse was *I* = 2.83 × 10^19^ W/cm^2^, and the power of the laser pulse was *P* = 144 TW with laser energy of *E*_*L*_ = 8.6 J. The driving laser pulse was launched into plasma that was homogeneous in the *x* direction and linearly polarized in the *y* direction.

### Radiation modeling

The angle-resolved spectrum of the betatron radiation emitted from a single electron in the vertical direction can be written as^12^


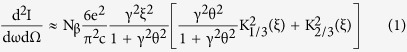


where *ξ *= *ω*/*ω*_*c*_(1 + *γ*^2^*θ*^2^)^3/2^, *ω*_*c*_ = 3*Kγ*^2^*ω*_*β*_, and *K*_1/3_ and *K*_2/3_ are modified Bessel functions of the second kind. For *θ* = 0, the on-axis spectrum of the betatron radiation is given by


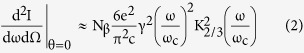


For an electron beam, the on-axis spectrum is an integration of contributions from all the electrons in the beam which can be written as


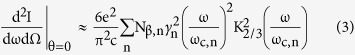


where *N*_*β,n*_ is the number of periods of betatron oscillation performed by the n^th^ electron, *γ*_*n*_ is the Lorentz factor of the n^th^ electron and *ω*_*c,n*_ is the critical frequency of the radiation emitted from the n^th^ electron. Here the acceleration process was not taken into account since most of the betatron radiation photons are emitted when electrons reaches to their maximum energy. Therefore, the electron spectra when electron bunches reach to their maximum energy were used to calculate the on-axis spectrum of the radiation. The parameters of *N*_*β*_, *γ* and *ω*_*c*_ are determined as follow: For each electron, *γ* was given by the electron spectra; *ω*_*c*_ was given by its *γ* and oscillation amplitude *r*_*β*_. The betatron amplitude *r*_*β*_ was obtained from the statistic on the betatron amplitude of each betatron period from all electron trajectories. For the first electron bunch, the average *r*_*β*_ = 1 μm, with the root mean square (rms) error of 0.3 μm, while for the second electron bunch, the average *r*_*β*_ = 9 μm, with rms error of 2.9 μm. Note that the betatron amplitude hardly depends on the electron energy, as we can see from Fig. 3a–c for the first bunch and Fig. 3d–f for the second bunch, respectively. So, we take the betatron amplitudes as constant for both electron bunches. *N*_*β*_ was also chosen as constant by averaging the electron trajectories which gives *N*_*β*_ = 10 and 15 for the first and the second electron bunch.

The distribution of the on-axis spectrum of the radiation emitted from a single electron satisfy the function of *F*(*ξ*) = *ξ*^*2*^*K*^*2*^_*2/3*_(*ξ*). In principal, the integrated spectrum would definitely deviates from the *ξ*^*2*^*K*^*2*^_*2/3*_(*ξ*) distribution as long as the energy of all electrons in the beam is not identical. However, for the second electron bunch, although the energy spread is large, the *ξ*^*2*^*K*^*2*^_*2/3*_(*ξ*) distribution was still used to fit the integrated spectrum, and the best fitting is not deviates much, as shown in Fig. 4, due to the fact that the high energy tail of the electron spectrum contributes most. The best fitting gives a critical energy of *E*_*c*_ ~ 1.2 MeV.

## Additional Information

**How to cite this article**: Ma, Y. *et al.* Generation of femtosecond γ-ray bursts stimulated by laser-driven hosing evolution. *Sci. Rep.* **6**, 30491; doi: 10.1038/srep30491 (2016).

## Supplementary Material

Supplementary Video 1

Supplementary Information
