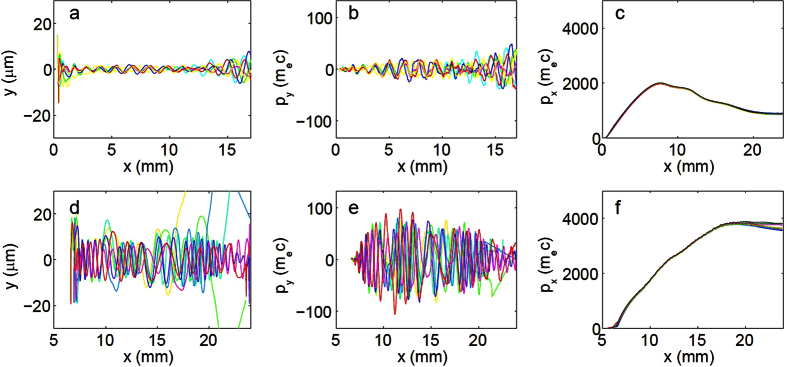# Erratum: Generation of femtosecond γ-ray bursts stimulated by laser-driven hosing evolution

**DOI:** 10.1038/srep32254

**Published:** 2016-09-02

**Authors:** Yong Ma, Liming Chen, Dazhang Li, Wenchao Yan, Kai Huang, Min Chen, Zhengming Sheng, Kazuhisa Nakajima, Toshiki Tajima, Jie Zhang

Scientific Reports 6: Article number: 3049110.1038/srep30491; published online: 07 26 2016; updated: 09 02 2016

This Article contains errors in Figure 3a and 3d where the y axes ‘y (μm)’ is incorrectly given as ‘y (mm)’. The correct Figure 3 appears below as Figure 1.

## Figures and Tables

**Figure 1:**