# Supplementary material for: Generation of femtosecond γ-ray bursts stimulated by laser-driven hosing evolution
Source: Sci Rep. 2016 Jul 26;6:30491. doi: 10.1038/srep30491 (PMC4960617; doi:10.1038/srep30491)
Supplement: Supplementary Information [file srep30491-s2.pdf]

Generation of femtosecond γ-ray bursts stimulated by laser-driven hosing evolution

Yong Ma, Liming Chen, Dazhang Li, Wenchao Yan, Kai Huang, Min Chen，
Zhengming Sheng, Kazuhisa Nakajima, Toshiki Tajima, Jie Zhang

Video legend:

A video of the evolution of the plasma density distribution and the laser field E_y is provided. (Top) the whole process of the self injection of the first bunch and the continuous injection of the second bunch as well as its transverse betatron oscillation. (Bottom) the corresponding evolution of the laser field E_y. The laser and plasma parameters used in the video are the same as that in Figure 1 in the manuscript.
